# Multidimensional screening and intervention program for neurocognitive disorder in vascular and multimorbid outpatients: Study protocol for a randomized clinical trial

**DOI:** 10.1371/journal.pone.0306256

**Published:** 2024-07-10

**Authors:** Cira Fundarò, Nicolò Granata, Silvia Traversoni, Valeria Torlaschi, Roberto Maestri, Marina Maffoni, Paola Baiardi, Federica Grossi, Michelangelo Buonocore, Paola Gabanelli, Marina Rita Manera, Antonia Pierobon

**Affiliations:** 1 Istituti Clinici Scientifici Maugeri IRCCS, Neurophysiopathology Unit of Montescano Institute (PV), Pavia, Italy; 2 Istituti Clinici Scientifici Maugeri IRCCS, Psychology Unit of Montescano Institute (PV), Pavia, Italy; 3 Istituti Clinici Scientifici Maugeri IRCCS, Department of Biomedical Engineering of Montescano Institute (PV), Pavia, Italy; 4 Istituti Clinici Scientifici Maugeri IRCCS, Direzione Scientifica Centrale of Pavia Institute, Pavia, Italy; 5 Istituti Clinici Scientifici Maugeri IRCCS, Psychology Unit of Pavia Institute, Pavia, Italy; Federal University of Paraiba, BRAZIL

## Abstract

**Background:**

The heightened risk of dementia resulting from multiple comorbid conditions calls for innovative strategies. Engaging in physical and cognitive activities emerges as a protective measure against cognitive decline. This protocol aims to discuss a multidomain intervention targeting individuals with dementias secondary to cerebrovascular or other medical diseases, emphasizing an often underrepresented demographic.

**Methods:**

This study primary objectives are: a) to identify patients affected by *Neurocognitive disorder due to vascular disease or multiple etiologies* (*screening and diagnostic phase*) and b) to evaluate the effectiveness of distinct rehabilitation protocols (*intervention phase*): motor training alone, paper-based cognitive rehabilitation combined with motor training, digital-based cognitive rehabilitation coupled with motor training.

**Discussion:**

Identifying cognitive impairment beyond rigid neurological contexts can facilitate timely and targeted interventions. This protocol strives to address the complex interplay of cognitive decline and comorbidities through a multidimensional approach, providing insights that can shape future interventions and enhancing overall well-being in this vulnerable population.

**Trial registration:**

The study has been registered on July 13, 2023 with the ClinicalTrials.gov NCT05954741 registration number (https://classic.clinicaltrials.gov/ct2/show/NCT05954741).

## Introduction

Dementia is an age-related clinical disease that leads to the impairment of mental functioning, activities of daily living, and behavior [1]. The World Health Organization (WHO) estimates that each year, 10 million individuals are newly diagnosed with dementia, affecting a global total of 50 million [1]. Recent evidence suggests that additional comorbidities can accelerate cognitive decline in those diagnosed with dementia [2,3]. However, limited research has been conducted on changes in dementia prevalence and incidence in comorbid populations [4,5]. In a longitudinal study spanning four years and involving a substantial sample of patients with comorbid conditions (2176 patients, 86.6% having more than two comorbidities), 37% developed mild cognitive impairment (MCI) or dementia [6].

Dementias secondary to cerebrovascular or other medical diseases are therefore of significant epidemiological and clinical relevance [7]. According to the Diagnostic and Statistical Manual of Mental Disorders (DSM-5-TR), these forms of Dementia are classified as *Neurocognitive disorder due to vascular disease or due to multiple etiologies* [8]. On this theme, epidemiological investigations have, for instance, identified Vascular Dementia as the second most prevalent origin of elderly dementia, constituting 15–20% of all dementia cases [9]. Research involving case-control methodologies has, additionally, pinpointed diverse risk elements associated with Vascular Dementia, encompassing factors such as advancing age, hypertension, diabetes mellitus, hyperlipidemia, recurrent stroke, cardiac issues, smoking, sleep apnea, and more recently, elevated levels of hyperhomocysteinemia, among other variables [9].

This finding holds significant implications within the realm of Public Health, as prevention and timely treatments stand to potentially reduce the future dementia case count among the elderly population [9]. As a result, the management of these patients should involve early diagnosis, effective treatment, and patient-centered care planning, both in specialized [10] and non-specialized settings [11–13].

Regarding treatment, it is well known that physical exercise can improve various aspects of health, including resistance, balance, strength, and cognitive functions such as attention and executive performance [14,15]. Additionally, cognitive rehabilitation appears to be an essential component of care, to be integrated with pharmacological and motor rehabilitation approaches for both the neurodegenerative condition and comorbidities, when present [14–17]. Moreover, novel technological methods and home-based interventions have been implemented within the framework of cognitive rehabilitation for patients with dementia [18–22]. A multidomain intervention could thus enhance cognitive functioning in elderly individuals with multiple morbidities [23,24].

However, despite advances in diagnosis and treatment, the current dementia care pathway remains suboptimal, lacking standardization and efficiency [11,13,25], and efficacy studies concerning the combination of cognitive and motor approaches to rehabilitation in this population are still not conclusive [26]. This aspect is likely influenced by the frequency of studies that prioritize intervention assessment in neurodegenerative forms of dementia and the lack of meticulous identification of this highly heterogeneous population during medical visits or scientific trials [27,28].

The protocol we present is a healthcare intervention project aimed at identifying individuals with early-stage cognitive deficits secondary to comorbidities in a multispecialty outpatient setting. Individuals identified as per the study criteria will be part of a rehabilitation program involving remote intervention through motor and cognitive rehabilitation programs. The effectiveness of this intervention will be assessed using multidimensional measures.

Considering the epidemiological importance of dementia forms stemming from vasculopathy and other organic conditions, the study aims to address unexplored nuances regarding interventions in this population. Thus, this protocol has two primary objectives: the first goal is to identify individuals with *Neurocognitive disorder due to vascular disease or due to multiple etiologies*, accomplished during a screening phase in an outpatient multidisciplinary environment.

The second aim involves testing, by means of a Randomized Controlled Trial (RCT), the efficacy of three distinct rehabilitation intervention protocols aimed at enhancing cognitive functions in patients previously diagnosed.

## Methods

### Trial design

This study is designed as an experimental randomized clinical trial, comprising two distinct phases: a screening and diagnostic phase and an intervention phase. During the *screening and diagnostic phase*, outpatients from neurological departments will undergo a comprehensive screening within a multidisciplinary outpatient setting. A set of diagnostic procedures will be employed to identify patients with *Neurocognitive disorder due to vascular disease or due to multiple etiologies*. Upon identification, patients meeting the study’s specific requirements will be enrolled. Subsequently, they will be guided to actively participate in the rehabilitation program delineated within this study’s intervention phase.

Our focus in the *intervention phase* will be an examination of the efficacy of three distinct rehabilitation protocols, namely: *motor rehabilitation alone*, *paper-based cognitive rehabilitation combined with motor rehabilitation and digital-based cognitive rehabilitation combined with motor rehabilitation*. Detailed descriptions of these protocols will be provided subsequently. Throughout the study, we are committed to adhering to the SPIRIT (Standard Protocol Items: Recommendations for Interventional Trials) guidelines to ensure methodological rigor and transparency in our protocol (S1 Appendix) [29].

### Study setting

The study will be conducted at two specialized medical centers renowned for rehabilitation and a multidisciplinary approach: Istituti Clinici e Scientifici Maugeri IRCCS Pavia, Italy; Istituti Clinici e Scientifici Maugeri IRCCS Montescano, Italy.

### Eligibility criteria

To be eligible for participation, individuals must meet the following criteria: a diagnosis of *Neurocognitive disorder due to vascular disease or due to multiple etiologies*, a Clinical Dementia Rating Scale (CDR) [30] score of 0.5 or 1, symptom onset within 12 months, an age range of 65 to 80 years, and the provision of written and signed informed consent indicating willingness to participate in the study.

The choice to implement the CDR scale reflects the intention to highlight the level of impairment in recruited patients. Recruiting subjects in the MCI or mild dementia phase is essential for the screening and preventive perspective of this trial. This is why included patients should be involved in an early phase of disease development and therefore reasonably capable of following the intervention program (e.g. using a tablet device, etc.).

### Exclusion criteria

Participants will be excluded if they meet any of the following criteria: presence of other known neurological conditions affecting cognitive function (such as Neurodegenerative Primary Dementia, Parkinson’s disease, Multiple Sclerosis, head trauma, or alcohol abuse), severe organic instability, ongoing neoplastic processes, severe psychiatric conditions, illiteracy, significant perception deficits, severe motor disabilities, specific intellectual deficits, engagement in other forms of training or neurostimulation within the past 6 months, or recent pharmacological interventions relevant to neurology within the month prior to the study.

### Objectives

The primary objectives of this study protocol encompass two key aspects:

1. S*creening and diagnostic phase* (delineation of the experimental population). The initial goal revolves around the comprehensive identification of patients affected by *Neurocognitive disorder due to vascular disease or multiple etiologies*. This objective will be primarily realized by undertaking a baseline neuropsychological in depth evaluation [31];
2. *Intervention phase* (assessment of rehabilitation protocol effectiveness). The subsequent focus of the study entails the evaluation of the efficacy associated with three distinct rehabilitation protocols (*Motor rehabilitation only*, *Paper-pencil cognitive intervention with motor rehabilitation*, *Digital cognitive intervention with motor rehabilitation*).

The exploration of other dimensions constitutes a secondary objective (See Table 1 for a more comprehensive measurement description). This encompasses an evaluation of motor skills, mood and anxiety levels, quality of life, and patient adherence to treatment. Additionally, this study will delve into the significance of effective communication in patient management, the burden experienced by caregivers, and the usability of digital devices when integrated into the treatment regimen.

**Table 1:** Protocol measures description.

**Screening measures**			
**Measure**	**General domain**	**Specific domain(s)**	**Description (task, evaluation)**
**General Practitioner assessment of Cognition (GPCog)** [32]	Cognitive performance	Orientation Memory Attention Language Visuospatial abilities	A cognitive impairment screening questionnaire tailored to evaluate cognitive function and pinpoint potential instances of dementia among older adults. This tool is frequently employed in primary care settings and can be administered by general practitioners or other healthcare professionals. The overall score spans from 0 to 9, with a score of 9 indicating normalcy, a score ranging from 5 to 8 being categorized as borderline, and a score falling below 5 classified as pathological.
**Clinical Dementia Rating (CDR) scale** [30]	Cognitive performance	Memory Orientation Judgment Problem Solving Community Affairs Home care Hobbies Personal Care	A commonly utilized tool to evaluate the extent of dementia and cognitive decline in individuals with Alzheimer’s disease and related conditions. The Clinical Dementia Rating (CDR) scale finds frequent application in both clinical and research contexts, allowing the tracking of dementia progression over time. In this assessment, the interviewer assigns a rating to the person’s impairment level in each domain on a scale of 0 to 3: 0 = No impairment; 0.5 = Questionable; 1 = Mild; 2 = Moderate; 3 = Severe.
**Cumulative Illness Rating Scale (CIRS)** [33]	Comorbidity level	Organ systems assessment Severity rating	Among the limited array of standardized tools available, this instrument facilitates physicians in providing assessments of health status across diverse dimensions. This measure entails clinical evaluations of the extent of pathology and impairment across 12 major organ categories, as well as within the psychiatric and behavioral realms. The scoring procedure entails evaluating the severity of medical conditions across various bodily systems and then aggregating the individual severity ratings to compute the overall Comprehensive Illness Rating Scale (CIRS) score.
**Outcome measures**			
**Measure**	**General domain**	**Specific domain(s)**	**Description (task, evaluation)**
**Mini-Mental State Examination (MMSE)** [39]	Cognitive performance	Orientation Memory Attention Language Visuospatial abilities	Assessment of diverse cognitive domains involves responding to 30 items that encompass spatial/temporal orientation, repetition and recall of three words, working memory (backward calculation and/or spelling), sentence repetition, sentence writing, naming, three-step command execution, and constructional praxis. The achievable score varies between 0 and 30, with patients scoring ≤23 indicating cognitive deficits, and scores ≤18 signifying a moderate level of dementia.
**Trail Making Test—A (TMT-A)** [44]	Cognitive performance	Visuospatial abilities Psychomotor speed Selective attention Selective memory	Linking 25 consecutive numbers on a sheet in ascending order as quickly as possible. The score is determined based on the number of seconds it takes to complete the task.
**Timed Up and Go (TUG)** [41]	Motor performance	Fall risk Static balance Dynamic balance	Rising from a chair, walking a distance of three meters, executing a turn, walking back to the chair, and resuming a seated position. The threshold for the total time taken in the test varies based on the specific clinical population under observation.
**6 Minutes Walking Test (6MWT)** [40]	Motor performance	Walking length	Walking independently (or assisted by an operator, if needed) along a straight corridor for a duration of 6 minutes. Subsequent to the test, the distance covered is computed.
**Basic Activities of Daily Living (BADL)** [42]	Functional performance	Basic daily activities	Assessment of six fundamental activities: bathing, dressing, toileting, continence, mobility, and feeding. A score of "0" signifies dependency on the respective activity, while a score of "1" indicates independence. The cumulative score is determined by adding the points assigned to each item.
**Instrumental Activities of Daily Living (IADL)** [43]	Functional performance	Levels of autonomy in life activities (basic and instrumental)	Evaluation of eight complex activities: using the telephone, shopping, using transportation, cooking, doing housework, doing laundry, handling money, and taking medications. A score of "0" is assigned if the subject is dependent on the individual activity and a score of "1" if independent. The total score is assigned as the sum of the scores obtained in all items.
**General Anxiety Disorder 7 (GAD 7)** [45]	Psychological variables	Anxious symptoms	Self-reported evaluation of the severity of anxious symptoms in the previous two weeks. The range is from 0 to 21, where scores of 5, 10, and 15 are considered cut-offs for mild, moderate, and severe anxiety, respectively.
**Patient Health Questionnaire-9** **(PHQ-9)** [46]	Psychological variables	Depressive symptoms	Self-reported evaluation of the severity of depressive symptoms in the previous two weeks. The scores range from 0–27: 0–4 = Absent; 5–9 = Subthreshold depression; 10–14 = Mild major depression; 15–19 = Moderate major depression; ≥ 20 = Severe major depression.
**EuroQoL 5D-5L** **EuroQoL VAS** [47]	Health-Related Quality of Life	Quality of life	Available in two different versions: 5D-5L: Self-reported evaluation of the difficulty level in motor abilities, personal care, usual activities, and the severity level of pain, anxiety, and depression. The patient evaluates the perceived level of impairment with a 3-points scale: 1, no problem; 2, moderate problem; 3, severe problem. VAS: Self-reported overall level of health. The visual-analogue scale is built on a thermometer model graduated from 0 (worst possible health condition) to 100 (best possible health condition).
**Neuropsychiatric Inventory (NPI-Q)** [49]	Psychological variables	Neuropsychiatric symptoms Caregiver burden	A caregiver-administered evaluation to appraise the presence and intensity of neuropsychiatric symptoms in patients across five key domains (depression, apathy, agitation, psychosis, aggression) during recent weeks, along with the ensuing impact on perceived stress levels. The severity of symptoms is gauged on a 3-point Likert scale (1 = slight, 2 = moderate, 3 = severe), while the caregiver’s stress rating scale employs a 6-point classification (0 = Absent; 1 = Minimal; 2 = Mild; 3 = Moderate; 4 = Severe; 5 = Very Severe or Extreme).
**Family Strain Questionnaire—Short Form (FSQ-SF)** [48]	Psychological variables	Caregiver burden	A caregiver’s self-report assessment of their experience within the caregiving context. The form offers an overview of the caregiver’s emotional burden and categorizes scores based on escalating levels of significance: "Urgent," signifying an immediate requirement for referral to a specialist in the psychiatric or psychological field; "Seriously Recommended," indicating the need to propose referral to a psychologist for assessment and assistance; "Recommended," suggesting a psychological interview for the caregiver with a focus on addressing the observed rise in perceived stress level; "Not Recommended," implying self-sufficient and practical management of caregiver-related health concerns.
**Communication Assessment Tool (CAT)** [50]	Adherence and Healthcare communication	Physician’s communication effectiveness	Self-reported assessment of patient’s perceptions of the physician’s communication effectiveness after a single encounter. Respondents are asked to rate different dimensions of physician communication and interpersonal skills using a 5-point scale (1 = poor, 2 = fair, 3 = good, 4 = very good, 5 = excellent).
**Semi-structured qualitative interview** **(patients)**	Adherence and Healthcare communication	Treatment adherence	A concise telephone interview designed to evaluate a patient’s engagement and adherence with the study intervention. The interview will be structured around the following inquiries: *Did you follow the prescribed program on a daily basis*? *If yes*, *did you face any challenges*? *If so*, *what type*? *Is there any additional information you would like to provide*? *Do you plan to continue participating in the study*?
**Guided interview** **(caregivers)**	Adherence and Healthcare communication	Treatment adherence	A daily guided, brief interview to support caregivers during patient observation. The interview utilized close-ended questions, while also including an open-ended final question to capture valuable free data: *1*. *Did the patient complete the assigned treatment today*? *Yes/No (if no*, *explain why)* *2*. *If yes*, *did the patient complete the treatment for the prescribed duration*? *Yes/No (if no*, *explain why)* *3*. *Were there any problems during the treatment or did the patient or caregiver report anything of note*? *Yes (describe the problems or reports)/No*
**System Usability Scale (SUS)** [51]	System usability	Patient-perceived usability	Self-reported assessment of the degree of perceived usability as a result of using a wide variety of devices and systems. The total scores are between 0 and 100, the higher the scores, the higher the degree of perceived usability. The score ranges from A = excellent usability to F = poor usability, based on the normal distribution of the percentile range of mean scores.

### Data collection

#### Screening and diagnostic phase

The *screening and diagnostic phase* will take place at the specialist outpatient clinics of the participating centers: respiratory, sleep disorders, cardiological, nephrological, internal medicine, geriatric, and endocrinological departments.

**Fig 1:**
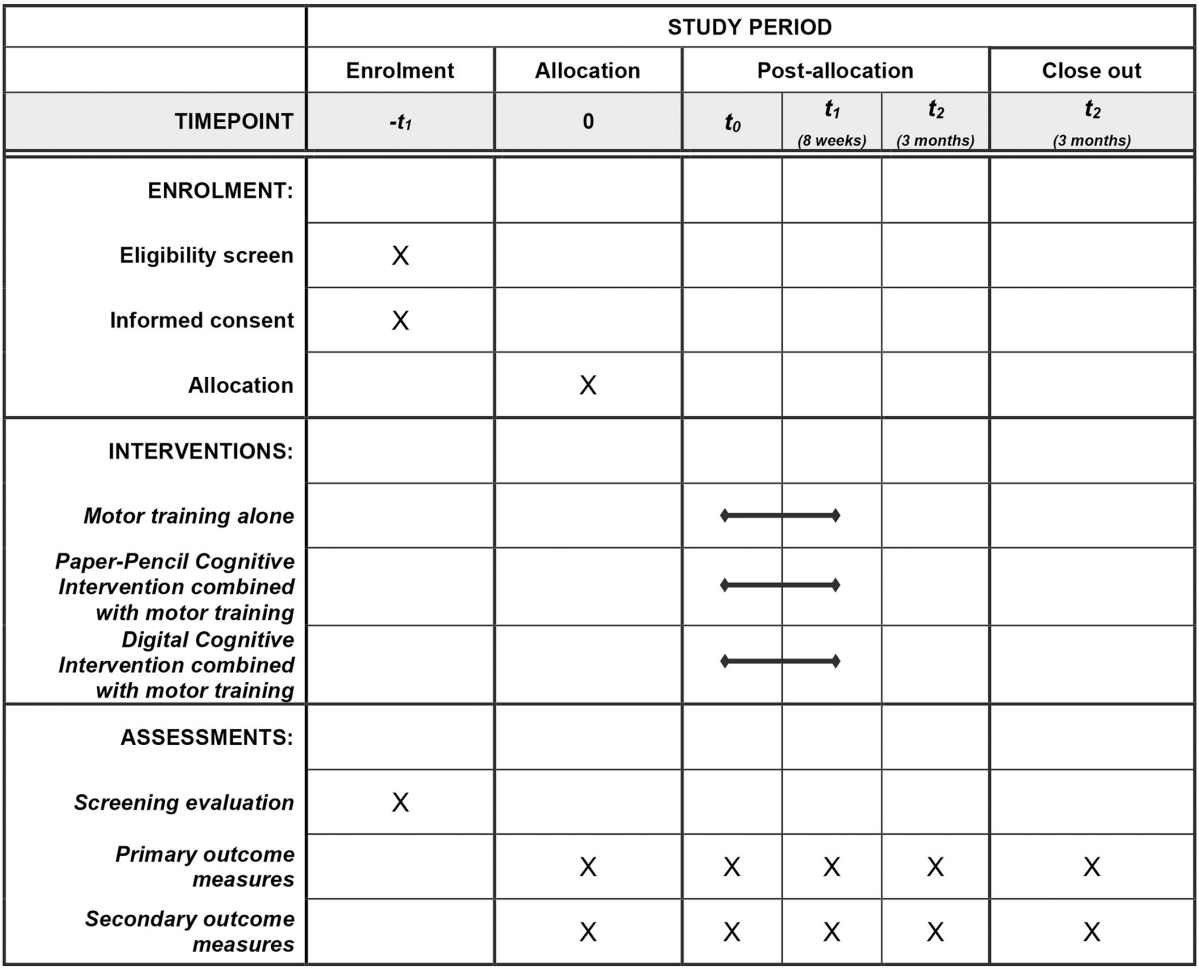
Schedule of enrollment, interventions, and assessments.

During routine specialistic visits (e.g. cardiological department) patients with suspected cognitive impairment (reported personally or by caregivers) will undergo a preliminary assessment composed by a routine medical examination and the administration of two screening measures, handled by the physician in charge: the General Practitioner Assessment of Cognition (GPCog), aiming to assess the global cognitive functioning [32] (see Table 1 and Fig 1) and the Cumulative Illness Rating Scale (CIRS), to assess the comorbidity level [33].

Outpatients with a GPCog score below 5 will be suggested to undergo a specialistic neurological and neuropsychological visit, conducted by the Neurophysiopathology and Psychology Units ICS Maugeri Pavia and Montescano, where additional assessment measures will be administered, in line with the most updated guidelines and to the current organizational care pathway [34,35].

Utilizing the data gathered during these visits, the neurologist will eventually identify and delineate a cohort of outpatients aligning with the DSM-5 diagnosis criteria for *Neurocognitive disorder due to vascular disease or multiple etiologies* [8]. Patients diagnosed will finally undergo a last assessment with Clinical Dementia Rating (CDR). The scale will be administered to assess the overall level of cognitive impairment. Patients presenting a CDR of 0.5 or 1 and meeting the study eligibility criteria, will be asked to join the intervention phase [30]. Each participant assessed will be asked to sign the agreement to the scientific analysis of the clinical data collected at the screening and diagnostic phase.

#### Intervention phase

The intervention phase itself will be conducted by the collaborative effort of the Neurophysiopathology, Psychology, and Neurorehabilitation Department at ICS Maugeri, spanning across Montescano (PV) and Pavia, Italy. Outcome measures will be administered at the Neurophysiopathology and Psychology Departments of ICS Maugeri in Montescano and Pavia (see Table 1).

After the assessment, patients willing to participate will be asked to sign a written informed consent in order to join the study. Consequently, participants will be randomly assigned to one of the three intervention groups (see the paragraph “Participants randomization” and Fig 1).

For motor rehabilitation, patients will be given precise instructions on the prescribed exercises by a physiotherapist during the first week at ICS Maugeri. In this initial week, the first session for each exercise will be supervised (pre-training). The pre-training is considered part of the intervention training. After this, patients will continue with the exercise routine independently on a weekly basis (see Table 2 for details).

**Table 2:** Weekly schedule per trial group.

		*Duration*	Day 1	Day 2	Day 3	Day 4	Day 5
			*Domain (s)*				
**Group 1** (90 min)	*Motor functions*	20 minutes	Fast-paced walking	Fast-paced walking	Fast-paced walking	Fast-paced walking	Fast-paced walking
		25 minutes	Balance and coordination	Postural control	Joint mobilization	Proprioception	Balance and coordination
		45 minutes	Muscle relaxation	Muscle relaxation	Muscle relaxation	Muscle relaxation	Muscle relaxation
**Group 2, Group 3** (90 min)	*Motor functions*	20 minutes	Fast-paced walking	Fast-paced walking	Fast-paced walking	Fast-paced walking	Fast-paced walking
		25 minutes	Balance and coordination	Postural control	Joint mobilization	Proprioception	Balance and coordination
	*Cognitive functions (paper-pencil or Khymeia VRSS Evo)*	45 minutes per day	Orientation Memory Attention/executive functions Visual-spatial functions	Memory Attention/executive functions Visual-spatial functions	Orientation Memory Attention/executive functions Visual-spatial functions	Memory Attention/executive functions Visual-spatial functions	Memory Attention/executive functions Visual-spatial functions

As for *cognitive rehabilitation* (including both paper-pencil and digital exercises), a process similar to that of motor exercises will be followed. In the initial weekly session, a psychologist will provide supervision. Following this, patients will undertake the exercise program on a weekly basis, adhering to a designated daily schedule of activities (Table 2).

Paper-pencil exercises will be curated by a neuropsychologist from a pool of options, carefully chosen in alignment with well-established neuropsychology standards [36,37].

Digital-based exercises will employ the Khymeia Virtual Reality Rehabilitation System (VRSS) Home Tablet [38] (see below). More in detail, the paper-pencil exercises are selected matching those offered by the Khymeia VRSS system, aiming to minimize uncontrollable biases and assess the effectiveness associated with different modes of exercise administration, while maintaining consistency in the exercise type/nature.

Participants across all three groups will undertake training sessions independently within their homes. The adherence to the training program will be assessed weekly through remote verification by both the physiotherapist and the psychologist. This will involve a semi-structured qualitative interview (see Table 1). Caregivers will play an observational role in the intervention, and they will be provided with comprehensive and standardized training sessions. The training will cover: recognizing and avoiding common biases, detailed instructions on how to observe and report behaviors and outcomes as well as techniques for maintaining consistency in observation and reporting. Furthermore, caregivers will be interviewed weekly to discuss patient adherence and any encountered challenge (Table 1).

#### Cognitive device system

The VRSS Home Tablet, developed by Khymeia Ltd. [38], is a Class I medical device that holds a CE marking, signifying compliance with European Union regulations for medical devices. This designation underscores the device’s safety and effectiveness in addressing specific medical conditions. The centerpiece of the VRSS is its Cognitive Module, which is meticulously designed to target neurological pathologies associated with cognitive functions. This module encompasses a range of activities meticulously curated to stimulate and rehabilitate cognitive processes. By leveraging the Virtual Reality (VR) potential, the VRRS provides a non-immersive experience, ensuring accessibility and comfort for users. This technology can be seamlessly integrated into a home-based rehabilitation program, allowing individuals to engage in tailored exercises from their own environment.

#### Patient allocation in groups

During the *intervention phase*, enrolled patients will be randomized and allocated into three groups (1:1:1 allocation ratio, see the paragraph “Participants randomization” and Fig 1), each undergoing related interventions of equal daily, weekly, and overall training duration (90 minutes total per day, 5 days per week, for a total of 8 weeks).

- Group 1—*Motor Training Alone*: Participants will engage in motor training, encompassing a 20-minute walking session, along with balance exercises, postural control exercises, proprioceptive exercises, joint mobilization exercises, and muscle strengthening exercises, collectively spanning 25 minutes. Subsequently, a 45-minute muscle relaxation session will follow, in order to reach the established 90 minute overall duration.
- Group 2—*Paper-Pencil Cognitive Intervention combined with motor training*: Participants will undertake motor training, mirroring Group 1 (45 minutes), supplemented by a standard cognitive intervention. This cognitive intervention involves executing paper-pencil exercises targeting attention, memory, executive function, visuo-spatial abilities, and space-time orientation (45 minutes total for the entire battery of paper-pencil exercises).
- Group 3—*Digital Cognitive Intervention combined with motor training*: Participants will perform motor training akin to Group 1 (45 minutes), in combination with a digital cognitive intervention (45 minutes total for the entire battery of digital exercises). This intervention comprises interactive cognitive exercises focused on attention, memory, executive functions, visual-spatial ability, and space-time orientation. The exercises will be facilitated by specific software and hardware (VRRS Home Tablet, Khymeia Srl).

### Outcome measures

#### Primary outcome measure

The primary measure implemented for the present study will be the MMSE (normed total) [39].

#### Secondary outcome measures

The assessment process will encompass various domains, including motor performance, functional performance, cognitive performance, health-related quality of life (HR-QoL), and psychological variables (mood and psychiatric assessment), caregiver burden, adherence, and health care communication and system usability (when the digital device was utilized). Regarding motor performance, we will administer the 6-minute walking test (6MWT) [40] and the Time Up and Go (TUG) test [41]. In terms of functional assessment, we will employ both the Basic Activities of Daily Living (BADL) [42] and the Instrumental Activities of Daily Living (IADL) [43]. In the cognitive domain, patients will primarily undergo the Trail Making Test (TMT-A) [44]. For psychological variables and quality of life, we will administer the following battery of assessments: Generalized Anxiety Disorder 7-Item Scale (GAD-7) [45], Patient Health Questionnaire-9 (PHQ-9) [46], and European Quality of Life (EuroQoL) [47]. Caregivers will complete the Family Strain Questionnaire (FSQ) [48],the Neuropsychiatric Inventory (NPI) [49], and a guided interview to support patients’ observation during daily exercises. In terms of adherence and communication related to health care management, patients will participate in a phone interview to assess program adherence (see Table 1) and complete the Communication Assessment Tool (CAT) [50]. Lastly, to evaluate the usability of the digital system utilized, we will administer the System Usability Scale (SUS) (Group 3, Digital Cognitive Intervention only) [51].

### Materials

See Table 1 for an extensive description of the instruments administered.

### Study timeline

**Fig 2:**
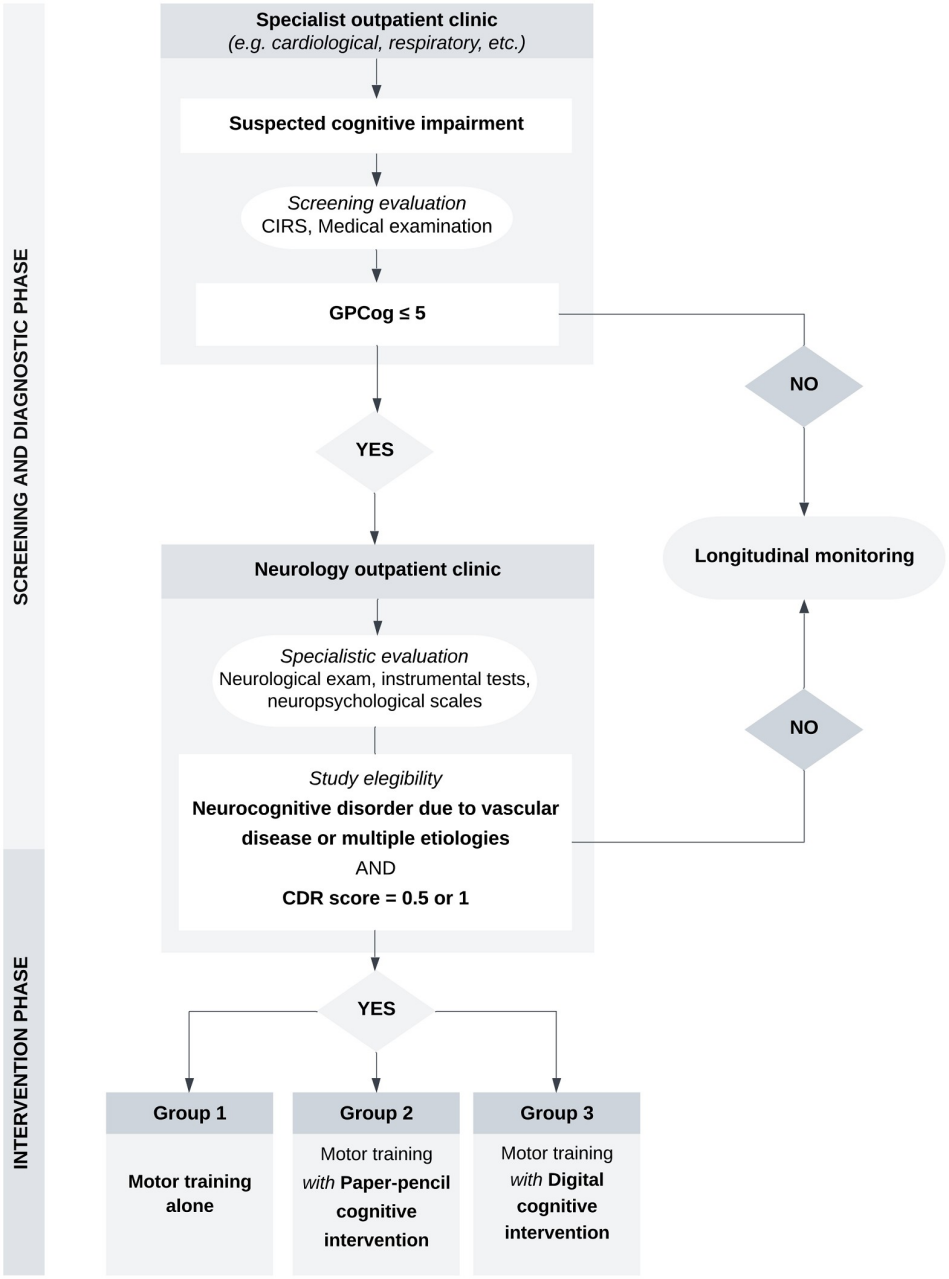
Study flowchart. GPCog, General Practitioner Assessment of Cognition; CIRS, Cumulative Illness Rating Scale; CDR, Clinical Dementia Rating Scale.

**T0 (Baseline):** The Neurophysiopathology and Psychology Units will collect and assess clinical and diagnostic data available at baseline, together with the inclusion and exclusion criteria, to proceed with the participant selection. Measures required by the study will be therefore administered (See Table 1). Participants will therefore be randomized into three groups (Figs 1 and 2).
**T1 (Post-training):** the outcome measures will be administered after 8 weeks of training.
**T2 (Follow-up):** the outcome measures will be furtherly administered 3 months after the end of the training.

### Study end-point

Effectiveness will be assessed according to the cognitive performances measured by MMSE [39]. As for the difference among groups, a difference (delta) of +1.4 will be hypothesized between the group *Digital-cognitive intervention combined with motor training* and the group *Paper-pencil cognitive intervention combined with motor training*, whereas a delta of +2.8 is expected to be observed between the subgroup *Digital-cognitive intervention combined with motor training* and the group *Motor intervention alone* [52].

### Data analysis

#### Sample size

The sample size was calculated considering the MMSE score, the primary outcome of the trial. We required the sample size to be large enough to detect a difference in efficacy (measured as MMSE at the end of treatment—MMSE at enrolment) of 1.4 MMSE points between the most effective treatment and the least effective treatment. This value was selected because it is reported in literature as the lowest threshold for the minimum clinically important difference (MCID) [39,53]. To be conservative, the third treatment was assumed to be as effective as the average of the other two. The aim was to detect this difference with a two-sided type I error of 5% and 80% power. From previous studies in populations similar to ours [54], the standard error of measurement (SEM) for the MMSE is 1 point, giving an SD of 1.41 (resulting in a Cohen’s effect size close to 1). The sample size was then estimated at 63 patients (21 per group). Taking into account the possibility of losing some patients (up to 16% of drop-outs), the required sample size was set at 75 patients (25 per group). Computations were performed using the ’proc power’ of the SAS statistical package with the following parameters: one-way ANOVA as statistical model, 0, 0.7 and 1.4 as group means, 1.41 as standard deviation, 0.05 as alpha and 0.8 as power (SAS/STAT, release 9.4, SA Institute Inc., Cary, NC, USA).

#### Statistical analysis

Data will be analyzed on the intention-to-treat (ITT) principle. Missing data will not be replaced. Prior to analysis, the normality of the data will be checked by the Shapiro-Wilks test, and an attempt will be made to convert non-normally distributed variables into normally-distributed variables using appropriate variable transformations (logarithmic transformation, square root transformation, reciprocal transformation, power transformation). Descriptive statistics will be reported as mean ± standard deviation for continuous variables and as N (percent frequency) for discrete variables. Between-group comparisons will be carried out by one-way analysis of variance (ANOVA) and by the Chi-squared test for continuous and categorical variables respectively. A significant result from ANOVA will be followed up by post-hoc analysis (Tukey HSD). The effect of treatment over time for the variables under consideration will be investigated by a two-factor repeated measures ANOVA, the first factor being TREATMENT (between factor, three groups: digital-based cognitive rehabilitation combined with motor rehabilitation, paper-based cognitive rehabilitation combined with motor rehabilitation, motor training alone), and the second factor being TIME (within factor, three measurements: baseline, post-treatment, and follow-up), with repeated measurements in the time factor. Mauchly’s test of sphericity will be used to assess the sphericity assumption and the Greenhouse-Geisser correction will be applied if appropriate. A significant result from repeated measures ANOVA will be followed up by post-hoc analysis for pairwise comparisons (Dunn-Sidak).

Between group baseline homogeneity of potential confounding variables should be guaranteed by the randomization protocol, but it will be checked before analysis. In case of significant differences, analysis of covariance (ANCOVA) will be used, adjusting for covariates that may influence the outcome.

All statistical tests will be two-tailed and a p-value <0.05 will be considered statistically significant. All analyses will be carried out using the SAS/STAT statistical package, release 9.4 (SAS Institute Inc., Cary, NC, USA) by a biostatistician from the Biomedical Engineering Unit.

#### Participants randomization

This will be a randomized parallel group trial with a 1:1:1 allocation ratio. Randomization will be performed using a block randomization algorithm (block size of 15) based on Matlab random number generator (MATLAB version: R2014a, Natick, Massachusetts: The MathWorks Inc) to ensure quantitative symmetry of patients assigned to the three groups even during enrolment and to allow for balanced interim analyses. Randomization will be centralized and performed by an independent biostatistician not involved in the trial, from the Biomedical Engineering Unit. Each patient will be given a unique patient number and a randomization code for the allocation at enrolment, so that the staff enrolling participants will know which treatment each patient will be assigned to only once the patient has been enrolled [55]. Finally, the biostatistician responsible for generating the randomization list and the biostatistician responsible for analyzing the data will be unaware of the association between randomization codes and treatment group. Furthermore, to ensure examiner blinding to the study condition, group allocation will be also concealed using alphanumeric codes assigned by a project manager after randomization. Group anonymization will minimize bias in the administration of study measures.

#### Informed consent

Each participant assessed will be asked to sign the agreement to the scientific analysis of the clinical data collected at the screening phase; participants enrolled in the intervention phase of the study will be asked to sign the written informed consent.

#### Withdrawal

Patients having acute organic events or manifesting the need for pharmacological treatment during the training will be excluded from the study.

Participants who no longer wish to participate in the trial may withdraw at any time without further explanation. To perform intention-to-treat analyses with as little missing data as possible, the investigator may ask the participant to use data already collected and whenever possible, the participant will be asked for permission to obtain data for the primary outcome measure.

#### Adverse events

Regarding safety considerations, one potential risk associated with participation pertains to the physiotherapy evaluation (TUG and 6MWT), as there is a possibility of falls. During the inpatient evaluation, physiotherapists will carefully follow the patients throughout the performances to intervene in case of need. To mitigate this concern at home, patients will be suggested to wear appropriate attire, such as closed-toe shoes to prevent sliding. Caregivers will be also provided with a leaflet including tips on how to prevent falls in a domestic environment. Cognitive adverse events are not expected [56].

#### Ethics and dissemination

The study design and the protocol were submitted and approved by the Institutional Review Board and by the Ethics Committee (Comitato Etico Istituti Clinici Scientifici ICS Maugeri Pavia, approval CE number 2618 date 9.3.2022) (S2 Appendix) and were implemented following the World Medical Association code of Ethics [57].

The trial database files will be anonymized and will be made available on ZENODO, a general-purpose open repository developed under the European OpenAIRE program.

## Discussion

Demographic factors and comorbid conditions increase the risk of dementia [58]. A recent systematic review identified numerous cognitive risk factors, with the majority (61.2%) related to comorbidities, particularly cardiovascular disease and diabetes [58]. Protective factors (20%) included a healthy diet, regular physical activity, and engaging in cognitively stimulating activities [59]. It is well-established that physical activity maintains neuronal integrity, preserves brain volume, and cognitive activity enhances neural circuit plasticity [60]. Physical activity and exercise interventions have demonstrated solid efficacy in dementia treatment, and as a result, these findings should be disseminated in primary and secondary care settings [61]. Cognitive stimulation has also been shown to significantly improve memory, activities of daily living, and depressive symptoms, although further confirmation is needed [62]. Despite the current absence of disease-modifying therapies, accurate programs for early dementia diagnosis and multidisciplinary interventions can provide appropriate care. Similarly, a recent review underscored the lack of a standard and accurate methodology in rehabilitation programs [26]. However, despite advancements in diagnosis, the current trajectory of dementia care remains suboptimal, characterized by insufficient standardization and inefficiency [11,13,23,25].

Concerning the efficacy of different rehabilitation programs, we generally expect a greater efficacy of combined motor-cognitive interventions compared to solely motor-based interventions [26]. As for digital rehabilitation, while other studies have already highlighted the efficacy of digital approaches in cognitive rehabilitation [20,63,64], it remains to be further discussed whether its implementation proves more effective than the traditional paper-pencil approach [23]. This necessitates a rigorous examination of both methods using standardized protocols [65]. The same statement is also applicable to the heterogeneity of efficacy results, and more specifically, to the target population. For all these reasons, it is worth investigating whether the lack of conclusive findings and the variability in study outcomes can be attributed, in part, to population definition.

In the present protocol we posit that a thorough categorization of distinct forms of dementia may provide valuable insights into the diverse impacts of multidimensional programs. Our objective is to strengthen currently available studies within this research thread by better refining the target population and by ascertaining the specific effect of a multimodal motor-cognitive training combination in rehabilitation.

### Limits, strengths, and project innovation

A potential limitation of the study is the use of the MMSE as the primary outcome measure. While the MMSE is a widely used and standardized cognitive screening tool in our institution, its sensitivity in detecting subtle cognitive changes is a matter of ongoing debate [66,67]. However, the MMSE offers several practical advantages that facilitated a faster and more efficient integration of this RCT into routine clinical practice. This streamlined approach potentially allows for a larger participant pool over time, which is crucial given the unexplored nature of this patient population. Furthermore, the MMSE’s well-established nature and standardized format provide several benefits. The extensive normative data available facilitates comparisons with past studies and across different populations. Additionally, staff familiarity with the MMSE minimizes variability in test administration. For future iterations of this RCT, incorporating more sensitive measures or specific cognitive batteries might be beneficial to capture subtle changes in cognitive function.

The absence of a non-interventional control group represents a second limitation of this study. However, given the intention to integrate the study into the standard workflow of our departments and the ethical considerations surrounding the withholding of standard care from participants, inclusion of such a control group was deemed impractical and potentially unethical. Another potential weakness of the project might be participant dispersion. Similarly, given the considerable number of outpatients to be screened, we expect the recruitment window to be time-consuming. On the other hand, the mixed methodology adopted for the intervention (a combination of motor and cognitive rehabilitation) as well as the continuity of care that it implies (home-based intervention) are indeed highlights of this study and might foster a generation of integrated rehabilitation protocols for cognitive impairment. Accordingly, the implementation of a longitudinal assessment with different outcome measurements will contribute to strengthening study results. Finally, the early detection of cognitive impairment in a non-strictly neurological environment might foster a forward intervention in the beginning stages of the disease.

## Conclusions

This study addresses the imperative need for innovative interventions in the realm of dementia care, specifically focusing on individuals burdened by dementias secondary to cerebrovascular or other medical diseases. Despite diagnostic strides, the existing care pathway continues to exhibit deficiencies in standardization and efficiency, underlining the urgency for novel approaches [68]. By discussing a multidomain intervention that encompasses both physical and cognitive rehabilitation, this research endeavors to bridge critical gaps in patient management. As we navigate the complexities of dementia care, early detection outside of traditional neurological contexts emerges as a pivotal aspect for tailored and patient-centered interventions. This pursuit aligns with the overarching objective of enhancing cognitive resilience and overall well-being in a vulnerable population that is frequently marginalized in the domain of dementia care.

## Supporting information

S1 Appendix SPIRIT checklist. (DOC)

S2 Appendix Approved study protocol. (DOCX)

## Abbreviations

6MWT: 6-minute walking test
BADL: Basic Activities of Daily Living
CAT: Communication Assessment Tool
CDR: Clinical Dementia Rating Scale
CIRS: Cumulative Illness Rating Scale
DSM 5 –TR: Diagnostic and Statistical Manual of Mental Disorders 5 –Text Revision
EuroQol: European Quality of Life
FSQ: Family Strain Questionnaire
GAD: Generalized Anxiety Disorder 7
GPCog: General Practitioner Assessment of Cognition
IADL: Instrumental Activities of Daily Living
MCI: Mild Cognitive Impairment
MMSE: Mini Mental State Examination
NPI: Neuropsychiatric Inventory
PHQ-9: Patient Health Questionnaire-9
RCT: Randomized Controlled Trial
SUS: System Usability Scale
TMT-A: Trail Making Test
TUG: Time Up and Go test
VRRS: Virtual Reality Rehabilitation System
