# Supplementary material for: Multidimensional screening and intervention program for neurocognitive disorder in vascular and multimorbid outpatients: Study protocol for a randomized clinical trial
Source: PLoS One. 2024 Jul 10;19(7):e0306256. doi: 10.1371/journal.pone.0306256 (PMC11236129; doi:10.1371/journal.pone.0306256)
Supplement: S2 Appendix — (DOCX) [file pone.0306256.s002.docx]

**Valutazione degli effetti di protocolli riabilitativi cognitivo-motori su pazienti affetti da decadimento cognitivo di origine vascolare o da altra condizione medica: studio randomizzato su pazienti selezionati mediante screening ambulatoriale multidisciplinare.**

Acronimo: RCT_CogDec_Rehab

**RATIONALE**

Numerous studies have highlighted that the risk of cognitive decline increases with the worsening overall organic condition of the individual. Multimorbidity, in fact, promotes the development of additional chronic pathologies and is also correlated with the risk of developing cognitive decline (mild cognitive impairment, MCI, or dementia). This condition appears to be oriented towards an increase in vulnerability and health risk. To indicate all this, the concept of a frail individual has been introduced, especially in the elderly, understood as a phenotype that includes: muscle weakness, motor slowing, fatigue, reduced activity, and weight loss; more recently, the concept of frailty has been expanded to include social and cognitive factors. The neurobiological mechanisms underlying cognitive frailty are considered to be vascular risk factors, metabolic conditions, nutritional status, hormonal factors, inflammatory state, and any associated psychosocial factors. Comorbidity and frailty increase the risk of evolving into a cognitive deficit at various levels of severity. Numerous studies have been conducted on the presence of comorbidities and the risk of cognitive decline with wide variability in terms of the prevalence of cognitive deficits. In one study on a large sample of comorbid subjects (2176 patients, of which 86.6% had >2 comorbidities) observed over a 4-year period, 37% developed MCI or dementia. Several studies have also assessed the relationship between physical frailty and the risk of developing cognitive frailty. The prevalence of cognitive frailty varies, according to the cases, from 1 to 5% in subjects with organic frailty. In subjects where the presence of a state of frailty is hypothesized, it is therefore necessary to establish a multidisciplinary assessment and intervention, including the analysis of clinical conditions, functional abilities, cognitive capacities, psycho-affective state, and pharmacological treatment of the individual. During the course of the disease, the patient with multiple frailty/comorbidity factors poses complex care problems that involve not only healthcare and social workers but also and above all family members. Physical exercise improves resistance, balance, and strength as well as cognitive functions such as attention and executive functions through a series of metabolic and neurochemical brain mechanisms. Cognitive intervention is an important addition to pharmacological treatment (both for defined neurodegenerative cognitive pathology and comorbidities if present), stimulating cognitive functions, although the evidence of effectiveness is not unequivocal and therefore needs further research.

There are initial indications that the combination of cognitive and motor training may improve or maintain cognitive functions more effectively than a single type of intervention. In the field of cognitive rehabilitation for individuals with dementia, models of home-based rehabilitative intervention have been proposed using technological supports (tablets equipped with cognitive rehabilitation software); this type of treatment shows promising results in maintaining the cognitive functioning of individuals in the early stages of the disease. In this study, individuals with early-stage neurocognitive decline of vascular/other medical etiology (DSM V) will be evaluated, undergoing various rehabilitation intervention models.

PRIMARY OBJECTIVE AND ENDPOINT

Primary objective of the study: The main goal of the study will be to evaluate the efficacy of different rehabilitation protocols on cognitive performance. Specifically, the effectiveness of a "technological" rehabilitation protocol will be assessed in comparison to other rehabilitation methodologies applied in the study.

Endpoint: The efficacy will be evaluated as the improvement in cognitive performance measured through the Mini-Mental State Examination (MMSE). Regarding differences between groups, a difference (delta) of +1.4 MMSE points is hypothesized between the "technological cognitive intervention group" and the "cognitive intervention with paper-and-pencil support group," and +2.8 between the "technological cognitive intervention group" and the "sole motor intervention group."

Secondary Objectives

They will include:

Improvement in distinct cognitive tests for each function.

Improvement in motor performance: distance covered in the 6-Minute Walk Test (6MWT), Timed Up and Go (TUG) score.

Improvement in mood tone and anxiety levels (GAD-7, PHQ-9), behavioral changes (NPI).

Evaluation of plasma biomarkers (baseline, post-training, and follow-up for Brain-Derived Nerve Factor BDNF).

Functional changes in brain function using functional MRI.

Changes in brain metabolism using PET Fluorodeoxyglucose (FDG).

Improvement in quality of life (EuroQoL).

Patient adherence to treatment (measured with MGL Adherence Scale).

Evaluation of the role of communication in patient management within the proposed pathway (Communication Assessment Tool - CAT).

Reduction of caregiver burden (Family Strain Questionnaire - FSQ).

**METHODS**

Study Type: Multicentric, low-intervention experimental randomized trial.

Study Sequence: The study will be preceded by a screening phase, followed by the intervention phase of the study:

Screening Phase: Aimed at identifying subjects with suspected cognitive impairment who attend the multi-specialist clinics at ICS Maugeri (neurological, pneumological, sleep disorders, cardiological, nephrological, internal medicine, geriatric, endocrinological in Pavia and Montescano) for their specific morbid conditions. Subjects reporting subjective cognitive disturbances will undergo clinical and instrumental diagnostic evaluation as per the PDTA ICS Maugeri diagnostic pathway (Fig.1). The diagnostic screening phase is detailed in Appendix 1.

In summary, patients with suspected cognitive decline will undergo diagnostic exploration through neurological evaluation, neuropsychology, and instrumental diagnostics (neuroimaging, nuclear medicine) to identify subjects with mild neurocognitive disorder (otherwise defined as Mild Cognitive Impairment, MCI) of vascular/other medical etiology (DSM V American Psychiatric Association 2013) with functional impairment assessed by Clinical Dementia Rating scale (CDR) ≤ 1 (initial phase) and major neurocognitive disorder (otherwise defined as cognitive deterioration) of vascular/other medical etiology (DSM V American Psychiatric Association 2013) with CDR ≤ 1 (initial phase).

Participation and scientific collaboration in the study are anticipated from the C. Mondino Neurological Institute in Pavia. Patients attending their neurological clinics will be evaluated diagnostically according to their own PDTA, consistent with our diagnostic process, to identify subjects with neurocognitive disorders resulting from comorbidities. If these patients meet the study criteria, they will be recruited at the Mondino Institute and then directed to the neurology clinic at ICS Maugeri for participation in the rehabilitation program outlined in the study.

All subjects evaluated at the neurology clinics of ICS Maugeri and the Mondino Institute will be asked to adhere to the research treatment upon receiving the results of the diagnostic screening phase.

All clinical data from the study will be collected in a dedicated database (RedCap Memory Clinic) (Attachment 1).

Intervention Phase

Within the neurology clinic at ICS Maugeri, subjects with the clinical conditions under study will be evaluated based on the study's inclusion/exclusion criteria. After expressing consent to participate in the research, they will be randomly assigned to one of three rehabilitation intervention groups described below (Fig.2).

Inclusion Criteria:

Cognitive decline, onset of symptoms < 12 months, age between 65 and 80 years, CDR ≤ 1, expression of informed consent to the study.

Exclusion Criteria:

Other known neurological pathologies affecting cognitive function (Parkinson's disease, multiple sclerosis, head trauma), history of alcohol abuse, severe organic instability (cardiorespiratory failure, hepatic, renal, or endocrine failure, ongoing neoplasm), diagnosis of severe psychiatric illness, illiteracy, severe sensory deficits preventing patients from undergoing neuropsychological screening tests and subsequently the proposed training, severe motor disability making it impossible to perform motor training, known intellectual disability, engagement in other forms of training/stimulation in the 6 months prior to the study, neurological pharmacological interventions in the month preceding the start of the study.

Patients enrolled in the intervention phase of the study, following a randomization model, will be divided into three groups and initiated into the intervention with equal daily training duration, weekly frequency, and overall training duration:

Group 1: Motor training;

Group 2: Motor training + "paper-and-pencil" cognitive intervention;

Group 3: Motor training + "technological" cognitive intervention.

If a patient experiences an acute organic event during training or requires neurological pharmacological intervention, they will be excluded from the study.

**Group 1:**

Subjects included in the study will undergo motor training consisting of: walking (20 minutes), balance exercises (10 minutes), muscle strengthening exercises (15 minutes) for a total of 45 minutes, followed by muscle relaxation exercises for another 45 minutes. The training will be conducted for 5 days per week for 12 weeks. The first treatment session will be in the presence of a physiotherapist, with subsequent weekly remote adherence checks. The remaining training sessions will be carried out at home with an exercise program on a card provided to the patient during the weekly visit. Patients will be instructed by a physiotherapist on the exercises to be performed in the week preceding the training.

**Group 2:**

Group 2 will undergo: Motor training with modalities similar to Group 1 (45 minutes)

"Standard" cognitive intervention through the execution of cognitive exercises related to attention, memory, executive functions, visuospatial skills, and orientation using paper and pencil (45 minutes). Participants will receive a "paper-and-pencil" exercise program selected by the neuropsychologist. The cognitive intervention includes a weekly individual treatment session, along with a conversation with the caregiver. In the "paper-and-pencil" treatment, exercises will be selected from specific manuals. The neuropsychologist will select the type and amount of material to assign to the patient for autonomous home training, with a timing divided by cognitive function.

**Group 3:**

Group 3 will undergo: Motor training with modalities similar to Group 1 (45 minutes)

"Technological" cognitive intervention through the execution of cognitive exercises related to attention, memory, executive functions, visuospatial skills, and orientation using a tablet with exercises provided by dedicated software (VRRS Home tablet Khymeia Srl) for 45 minutes. Participants will receive an exercise program selected by the neuropsychologist and performed at home. Each session will consider different cognitive domains. The entire training will be conducted for 5 days per week for 12 weeks. Sessions will be organized as follows: one weekly face-to-face treatment session (remotely). At the end of the session, a conversation with the caregiver will take place. The remaining 4 sessions will occur without the support of the neuropsychologist. In the face-to-face session, the daily division of activities to be performed and the level of difficulty of the exercises will be done weekly, starting from a baseline level and with potential weekly increments. Patients will be instructed by the neuropsychologist on the type of cognitive intervention with tablet support in tele-rehabilitation. The training will involve an initial in-person meeting for the delivery of the tablet (tablet usage training) and five remote sessions with the neuropsychologist (training for tele-rehabilitation). The caregiver will also participate as an observer during the instruction phase. The Virtual Reality Rehabilitation System (VRRS) developed by Khymeia will be used for the "technological" rehabilitation intervention.

Intervention Detail:

Day 1: 1 orientation exercise, 1 memory exercise, 2 attention/executive function exercises, 2 visuospatial function exercises;

Day 2: 2 memory exercises, 2 attention/executive function exercises, 2 visuospatial function exercises;

Day 3: 1 orientation exercise, 1 memory exercise, 2 attention/executive function exercises, 2 visuospatial function exercises;

Day 4: 2 memory exercises, 2 attention/executive function exercises, 2 visuospatial function exercises;

Day 5: 2 memory exercises, 2 attention/executive function exercises, 2 visuospatial function exercises.

Quantity: 6 exercises per day, each lasting 9 minutes.

**Table 1: Khymeia exercises**

| **DOMAIN** | **VRSS Exercise** |  |
| --- | --- | --- |
| **Orientation** | **Orientation** |  |
| **Memory** | **Library, Visual memory,**  **Word recall** |  |
|  |  |  |
| **Executive function/Attention** | **Farm, Attentive Matrices** |  |
|  |  |  |
| **Visuo spatial functions** | **Dots connection, Puzzle** |  |

**Outcome Measures of the Study**

Primary:

MMSE (corrected score)

Secondary:

Cognitive Tests: TMT A;

Motor Skills: 6MWT, TUG;

Functional Assessment: BADL, IADL;

Plasma Biomarkers: T0, T1, T2 BDNF;

Emotional Conditions (GAD-7, PHQ-9), Behavioral (NPI);

Adherence MGL Adherence Scale;

Communication CAT Communication Assessment Tool;

Quality of Life EuroQ;

Family Strain Questionnaire (FSQ);

System Usability Scale SUS (group 3 "technological" rehabilitation);

fMRI;

FDG PET.

Timing of the Intervention Phase (Table 2)

Time T0: In the Neurology Clinic of ICS Maugeri, clinical and diagnostic data collected up to this point are tabulated as clinical and anamnestic data and evaluated along with the inclusion/exclusion criteria to proceed with enrollment in the study following the expression of informed consent. The assessments specified by the study are then applied: MMSE, patient's motor skills (TUG timed up and go test, 6MWT), activities of daily living (ADL, IADL), CAT, MGL Adherence scale, EuroQoL; FSQ (caregiver); a blood sample is taken for the measurement of plasma biomarkers; randomization is carried out; subjects included are assigned to groups (1, 2, 3).

Time T1 after 12 weeks: application of outcome measures.

Time T2 after 3 months from the end of training: application of outcome measures.

Verification Methods for Study Timepoints (T0, T1, T2): in-person frontal visits;

Intra-phase verification methods: weekly remote visits for overall adherence to training, with particular reference to motor training; weekly in-person or remote frontal meetings for cognitive rehabilitation.

**Tab. 2 Project** **Timeline**

| **ATTIVITA’** | **SCREENING** | **T0** | **T1 (+ 12 weeks)** | **T2 (+ 3 Months)** |
| --- | --- | --- | --- | --- |
| Multidisciplinary outpatient clincis | X |  |  |  |
| Baseline neuropsychological tests | x |  |  |  |
| Neurology outpatient clinic | x |  |  |  |
| Diagnostic assessment (cognitive tests, RMN, PET) | x |  |  |  |
| Inclusion and exclusion criteria application |  | x |  |  |
| Informed consent/data |  | x |  |  |
| Randomization |  | x |  |  |
| Motor evaluation |  | x | x | x |
| Motor training |  | X groups 1,2,3 | - | // |
| Cognitive intervention |  | X groups 2,3 | - | // |
| Functional evaluation / outcome measures |  | x | x | x |

**ANALYSIS**

**SAMPLE SIZE**

The study aims to highlight, with a power of 0.80 and an alpha of 0.05, assuming a standard deviation of +1.4 MMSE points, an alternative hypothesis suggesting a difference (delta) between the means of the technological cognitive rehabilitation group compared to the "pen-and-paper" cognitive rehabilitation group (delta of +1.4 MMSE points) and against the motor rehabilitation-only group (+2.8 points). Therefore, 21 subjects per group will be required, totaling 63 subjects. To account for an approximate 20% dropout rate, the total sample size will be 75 subjects (25 per group).

**STATISTICAL ANALYSIS**

The collected data will be described using standard descriptive statistical techniques, including measures of central tendency and dispersion, frequencies, and percentages as appropriate. Normality of the data will be assessed using the Shapiro-Wilk test. Differences between groups at different time points will be examined through analysis of variance (ANOVA). Post hoc tests and Bonferroni correction will be applied if necessary. The statistical significance level will be set at p < 0.05.

**RANDOMIZATION**

Randomization will be carried out by assigning a consecutive number after patient enrollment and subsequent placement in a randomization list. Patients will be allocated to Group 1, 2, or 3 using a computerized program. Randomization will be performed by the Principal Investigator (PI).

**LIMITATIONS, STRENGTHS, AND INNOVATION OF THE PROJECT**

Weaknesses: Potential patient dispersion, and relatively long recruitment times considering the expected substantial number of patients undergoing screening.

Strengths/Innovations: Comprehensive intervention (clinical, motor, cognitive), generalizability of the intervention (tele-rehabilitation), longitudinal evaluation, and the use of different biomarkers as an outcome measure.

**Description of the Instruments Used in the Study**

**Mini Mental State Examination (MMSE) (Folstein et al., 1975):** It is a widely used screening test for dementia. It measures cognitive functions such as orientation, memory, attention, language function, and visuo-spatial skills. Scores range from 0 to 30; patients with scores ≤23 exhibit cognitive deficits, while scores ≤18 indicate moderate dementia.

For selective cognitive functions**, the Trail Making Test A** will be used as a secondary outcome measure (Giovagnoli et al., 1996). This test assesses visuo-spatial abilities, psychomotor speed, and selective attention. It involves connecting 25 numbers in ascending order in the shortest time possible. If the subject completes the first part of the test, the Trail Making Test B can be administered, where the subject connects numbers and letters alternately in the shortest time possible. The score is based on the seconds taken to complete the task.

**Timed Up and Go Test (Steffen et al., 2002):** This functional test evaluates the risk of falls, static and dynamic balance. The patient is timed while getting up from a chair, walking three meters, turning, walking back to the chair, and sitting down. The total time cutoff varies depending on the observed clinical population.

**Six Minute Walking Test (6MWT) (Holland AE et al., 2014):** This test assesses the patient's response to therapy in pulmonary and cardiovascular diseases. It is also used to measure the patient's functional status and predict morbidity and mortality. The patient walks in a straight corridor with a smooth surface for six minutes, and the distance covered is calculated.

**Basic Activity of Daily Living (BADL) (Katz, 1963):** This scale assesses basic daily activities, and the patient's independence is scored on a scale of 0 to 1 for six activities.

**Instrumental Activity of Daily Living (IADL) (Lawton and Brody, 1969):** It evaluates autonomy in instrumental daily activities, with scores ranging from 0 to 8 for women and 0 to 5 for men.

**Generalized Anxiety Disorder-7 (GAD-7) (Spitzer et al., 2006):** A questionnaire to measure the severity of anxiety symptoms in the last two weeks. Scores range from 0 to 21.

**Patient Health Questionnaire-9 (PHQ-9) (Spitzer et al., 1999):** This scale is used to determine the diagnosis, severity, and monitoring of depressive disorders. Scores range from 0 to 27.

**Neuropsychiatric Inventory (NPI-Q) (Kaufer et al., 1994): A** caregiver self-administered questionnaire to assess neuropsychiatric symptoms in the patient and the caregiver's stress level.

**EuroQoL:** Two scales measuring health-related quality of life using a questionnaire (EuroQoL 5d) and a visual analog scale (EuroQoL VAS).

**Family Strain Questionnaire (FSQ) – Short Form (FSQ-SF) (Silvia Rossi Ferrario, 2004/2008):** A screening tool to collect information on the situation experienced by a primary caregiver.

**Communication Assessment Tool (CAT) (Scala D. et al., 2016):** A questionnaire to assess patients' perception of the communicative effectiveness of the doctor.

**MGL Adherence Scale (Morisky DE et al., 1986):** A questionnaire widely used to assess non-adherence to pharmacological prescriptions.

**System Usability Scale (SUS):** A scale to evaluate the perceived usability after using various devices and systems, including technological equipment.

**fMRI:** Functional magnetic resonance imaging will be conducted using a 3T MRI machine to analyze resting-state brain activity and structural brain characteristics.

**Plasma Biomarkers (BDNF):** Serum levels of Brain-Derived Nerve Factor (BDNF) will be quantified to demonstrate their correlation with functional recovery.

**FDG PET:** PET imaging with fluorodeoxyglucose (FDG) to assess brain glucose metabolism.

VRRS Home Tablet: A Class I Medical Device for cognitive rehabilitation in virtual reality, allowing remote therapist-patient interaction.

These instruments will be employed to evaluate various aspects, including cognitive functions, motor skills, daily activities, emotional conditions, adherence, usability, and biomarkers, as part of the study's comprehensive assessment protocol.

**References**

-Multimorbidity and risk of mild cognitive impairment. Vassilaki M. J Am Geriatr Soc. 2015 Sept; 63(9): 1783-1790.

-Cognitive frailty in geriatrics. Arai H et al. Clin Geriatr Med 34 (2018) 667-675.

-Different cognitive frailty models and health and cognitive related outcomes in older age: from epidemiology to prevention. Panza F et al. Journal of Alzheimer’s disease 62 (2018)993-1012).

-Frailty screening and interventions: considerations for clinical practice. Walston J. Clin Geriatr Med. 2018 Feb ; 34(1): 25-38.

-Frailty, cognitive decline, neurodegenerative diseases and nutrition interventions. Gomez-Gomez ME. Int J Mol Sc. 2019, 20, 2842.

-Rockwood K et al A global clinical measures of fitness and frailty in elderly people. CMAJ 2005 173:489-495

-Effects of physical exercise on neuroinflammation, neuroplasticity, neurodegeneration and behaviour: what we can learn from animals models in clinical settings. Svensson M. Neurorehabilitation and neural repair. 2015, 29(6): 577-589.

-Brief cognitive screening instruments for early detection of Alzheimer’s disease: systematic review. De Roeck EE et al. Alzheimer’s research & therapy (2019) 11:21

-Diagnostic and statistical manual of Mental Disorders. American Psychiatric Association. 2013

-Cognitive training and cognitive rehabilitation for persons with mild to moderate dementia of the Alzheimer’s or vascular type: a review. Bahar-Fuchs A et al. Alzheimer’s Research & Therapy 2013, 5: 35.

-Positive effects of combined cognitive and physical exercise training on cognitive function in older adults with mild cognitive impairment or dementia: a meta-analysis. Karssemeijer EGA et al. Ageing Research Reviews 40(2017) 75-83.

-Effects of combined physical and cognitive exercises on cognition and mobility in patients with mild cognitive impairment: a randomized clinical trial. Shimada H et al. JAMDA 19 (2018) 584-591.

-Influence of combined physical and cognitive training on cognition: a systematic review. Lauenroth A et al. BMC Geriatrics (2016) 16: 141.

-Trevor Powell, Kit Malia Training di riabilitazione cognitiva. Esercizi di memoria, abilità di pensiero e funzioni esecutive dopo una lesione cerebrale. Erikson, 2009.

-Il training cognitivo per le demenze e le cerebrolesioni acquisite. Guida pratica per la riabilitazione. Iannizzi P, Bergamaschi S, Mondini S, Mapelli D. ed. Raffaello Cortina 2015

-Technology and dementia: the future is now. Astell AJ. Dement Geriatr Cogn Disord. 2019 Jul; 47(3): 131-139.

-Feasibility and efficacy of cognitive telerehabilitation in early Alzheimer’s disease: a pilot study. Jelcic N. Clinical Intervention in Aging. 2014; 9: 1605-1611.

-Effects of physical exercise on Alzheimer’s disease biomarkers: a systematic review of intervention study. Frederiksen KS et al. J Alzh Dis 61 (2018): 359-372

-Cerebral changes improved by physical activity during cognitive decline: a systematic review on MRI studies. Haeger A et al. Neuroimage: clinical 23 (2019) 101933

-Effect of 12-week home based cognitive training on cognitive function and brain metabolism in patients with amnestic mild cognitive impairment. Park J et al. Clinical intervention in Aging 2019: 14 1167-1175.

- Effectiveness of an innovative cognitive treatment and telerehabilitation on subjects with mild cognitive impairment: a multicenter, randomized, active-controlled study. Mantenti R et al. Front aging neurosci. 2020 Nov 16; 12: 585988. Doi: 10. 3389/fnagi. 2020. 585988.

-Technology-enhanced multi-domain at home continuum of care program with respect to usual care for people with cognitive impairment: the Ability-TelerehaABILITation study protocol for a randomized controlled trial. Realdon O, Rossetto F, Nalin M, Baroni I, Cabinio M, Fioravanti R et al. BMC Psychiatry. 2018 16: 425.

-Do cognitive interventions improve general cognition in dementia? A meta-analysis and meta-regression. Huntley JD et al. BMJ Open 2015; 5:e005247.doi: 10.1136/bmjopen-2014-005247).

-Kim J, Shin W. How to do random allocation (randomization). Clin Orthop Surg. 2014 mar; 6(1): 103-9).
